# Efficacy of Mobile Serious Games in Increasing HIV Risk Perception in Swaziland: A Randomized Control Trial (SGprev Trial) Research Protocol

**DOI:** 10.2196/resprot.6543

**Published:** 2016-11-22

**Authors:** Bhekumusa Wellington Lukhele, Patou Musumari, Christina El-Saaidi, Teeranee Techasrivichien, S. Pilar Suguimoto, Masako Ono Kihara, Masahiro Kihara

**Affiliations:** ^1^ Kyoto University Department of Global Health and Socio Epidemiology Kyoto University Kyoto Japan

**Keywords:** eHealth, mHealth, gamification, Internet, HIV prevention, innovation

## Abstract

**Background:**

The human immunodeficiency virus (HIV) and acquired immune deficiency syndrome (AIDS) continue to be a major public health problem in Sub-Saharan Africa (SSA), particularly in Swaziland, which has the highest HIV prevalence in this region. A wide range of strategies and interventions have been used to promote behavior change, though almost all such interventions have involved mass media. Therefore, innovative behavior change strategies beyond mass media communication are urgently needed. Serious games have demonstrated effectiveness in advancing health in the developed world; however, no rigorous serious games interventions have been implemented in HIV prevention in SSA.

**Objective:**

We plan to test whether a serious game intervention delivered on mobile phones to increase HIV risk perception, increase intention to reduce sexual partnerships, and increase intention to know own and partners HIV status will be more effective compared with current prevention efforts.

**Methods:**

This is a two-arm randomized intervention trial. We will recruit 380 participants who meet the following eligibility criteria: 18-29 years of age, own a smartphone running an Android-based operating system, have the WhatsApp messaging app, live in Swaziland, and can adequately grant informed consent. Participants will be allocated into a smartphone interactive, educational story game, and a wait-list control group in a 1:1 allocation ratio. Subsequently, a self-administered Web-based questionnaire will be issued at baseline and after 4 weeks of exposure to the game. We hypothesize that the change in HIV risk perception between pre- and post-intervention assessment is greater in the intervention group compared with the change in the control group. Our primary hypothesis is based on the assumption that increased perceived risk of HIV provides cues to engage in protective behavior. Our primary outcome measure is HIV risk perceived mean change between pre- and post-intervention compared with the mean change in the wait-list control group at 4-weeks post-intervention. We will use standardized regression coefficients to calculate the effect of the intervention on our primary outcome with *P* values. We will conduct both intention to treat and as treated analysis.

**Results:**

This study is funded by Hayao Nakayama Foundation for Science & Technology and Culture; Grant number H26-A2-41. The research and development approval has been obtained from Kyoto University Graduate School and Faculty of Medicine Ethics Committee, Japan, and Swaziland’s Ministry of Health Ethics and Scientific committee. Results are expected in February 2017.

**Conclusions:**

This study will provide evidence on the efficiency of a mobile phone interactive game in increasing HIV risk perception in Swaziland. Our findings may also be generalizable to similar settings in SSA.

**Trial Registration:**

University Hospital Medical Information Network Clinical Trial Registry ID number (UMIN-CTR):UMIN000021781; URL:https://upload.umin.ac.jp/cgi-open-bin/ctr_e/ctr_view.cgi?recptno=R000025103 (Archived by WebCite at http://www.webcitation.org/6hOphB11a).

## Introduction

It is estimated that 35.3 million people are living with human immunodeficiency virus (HIV) globally [1]. Sub-Saharan African (SSA) is the most affected region and the disease burden varies considerably between countries. In Swaziland, a land-locked, lower-middle income country surrounded by South Africa and Mozambique, HIV prevalence is estimated to be 26% among men and women of 15-49 years [2]. The overall HIV prevalence among the reproductive age population (18-49) has remained unchanged between 2006 and 2011 at 31% [3,4]. A recent, longitudinal, cohort study between December 2010 and June 2011 has estimated the incidence of HIV at 1.7% in men and as high as 3.1% in women [5]. Unprotected heterosexual transmission accounts for 94% of all new infections in the country [6]. More specifically, multiple concurrent partnerships have been identified as key drivers of HIV infection in Swaziland [6]. A recent qualitative study found that social and structural factors played a role in creating an enabling environment for high-risk sexual partnerships, and these factors included social pressure and norms, a lack of social trust, poverty and a desire for material goods, and geographical separation of partners [7].

Other key drivers have been highlighted in the Extended National Multi-sectoral HIV/AIDS Framework for 2014–2018 (eNSF) as: low rates of HIV testing (only 40% of people aged 15-49 had tested for HIV 12 months preceding a household survey) [8]; early sexual debut; low levels of medical male circumcision; and low HIV risk perception [8,9].

HIV is the leading public health concern in Swaziland [4]. National efforts have emphasized the scale-up of a combination of prevention approaches including: HIV testing and counseling, social behavior change communication, medical male circumcision, and HIV care and antiretroviral services. Despite this cocktail of prevention approaches, risky behaviors remain high. For example, Bicego and colleagues [4] note that there is still a low/late uptake of HIV testing services by men, which is consistent with late entry into care and treatment. Furthermore, according to the Swaziland Demographic Health Survey of 2006/07 and the Multiple Indicator Survey of 2010, the overall prevalence of multiple sexual partners remained unchanged at approximately 11% between 2006 and 2010 (data recalculated) [9,10]. On another note, the eNSF 2014-2018 points out that the Swaziland Social and Behavior Change strategy developed in 2010 has had limited success in facilitating desired levels of behavior change most importantly influencing personal HIV risk perception that focus on translating HIV awareness into protective action [8]. Beliefs about personal risk of HIV infection are central to motivate people to engage in behaviors that reduce their risk of HIV infection [11]. The Swaziland HIV testing and counseling guidelines includes HIV risk assessments to enhance self-perception of risk [12]. Models such as the Protection Motivation Theory and the Health Belief Model offer insights into the significance of perceived risk in adopting protective behavior. To date, there has been limited randomized control trials aimed at influencing how people perceived their risk of HIV in Swaziland.

Furthermore, anecdotal information suggests that there is information fatigue from the target audience in receiving HIV prevention messages from the mass media because most prevention campaigns have been dominantly delivered through mass media. One strategy that can break this perceived fatigue is the use of target audiences’ mobile phones. In developing countries, decreasing costs and increasing mobile network coverage provide a wide range of opportunities for apps using mobile phones [13]. Although comprehensive up to date data for mobile phone usage in Swaziland is limited, mobile phone penetration is estimated at 87% [14]. Our consultative meeting with the only mobile carrier in Swaziland revealed that there are currently 206,880 smartphones on the mobile network (as of June 2015). Therefore, our study seeks to use serious games delivered via mobile smartphones to engage the target audience in creative ways to increase personalization of HIV risk.

In this study, we adopt the definition of Serious Games as proposed by Alvarez and Djaouti [15], “a computer application whose intended purpose is to coherently combine both serious aspects such as, but not limited to teaching, learning, communication, or information, with game playing aspects from video games.” These combined serious aspects and playing aspects form a utilitarian scenario, which in computer terms uses a sound and graphics package, a story and appropriate rules, and is therefore distinct from simple entertainment [15]. Alvarez and Djaouti [15] summarize this definition by the following relationship:

Utilitarian scenario + gaming scenario => Serious Games.

Current literature suggests that serious games are effective in changing behavior. For example, a randomized trial (in the United States) designed to improve treatment adherence among 13- to 29-year-old patients with malignancies including acute leukemia, lymphoma, and soft-tissue sarcoma found that among 200 participants who were prescribed oral trimethoprim-sulfamethoxazole and 6-mercaptopurine, 16% indicted an increase in adherence for the serious games intervention group compared with the control group. Mixed-effect linear model analyses of chemotherapy metabolite concentrations showed that patients in the intervention group maintained significantly higher chemotherapy metabolite levels over time than did patients in the control group (significant group × time interaction; *P*=.002)[16]. Additionally, another clinical trial conducted in the United States among 935 males who had sex with males between 18- and 24-years old aimed at reducing risky sexual behavior and sexual shame, found that exposure to a serious games intervention led to immediate shame reduction for those in the serious games intervention group compared with the control group (mean [M]=−0.08, standard deviation [SD]=0.51, n=437 compared with M=0.07, SD=0.54, n=484, respectively; the difference was statistically significant at *t*_(919)_=4.24, *P*< .001) [17]. Despite the success of serious games in advancing health, no randomized intervention trials have been conducted in HIV prevention in SSA or in Swaziland. To address these research gaps, our goal is to design a serious game to increase HIV risk perception and use randomization to evaluate the efficacy of this intervention among 18- to 29-year-old people in Swaziland.

## Methods

### Study Design and Hypotheses

**Figure 1:**
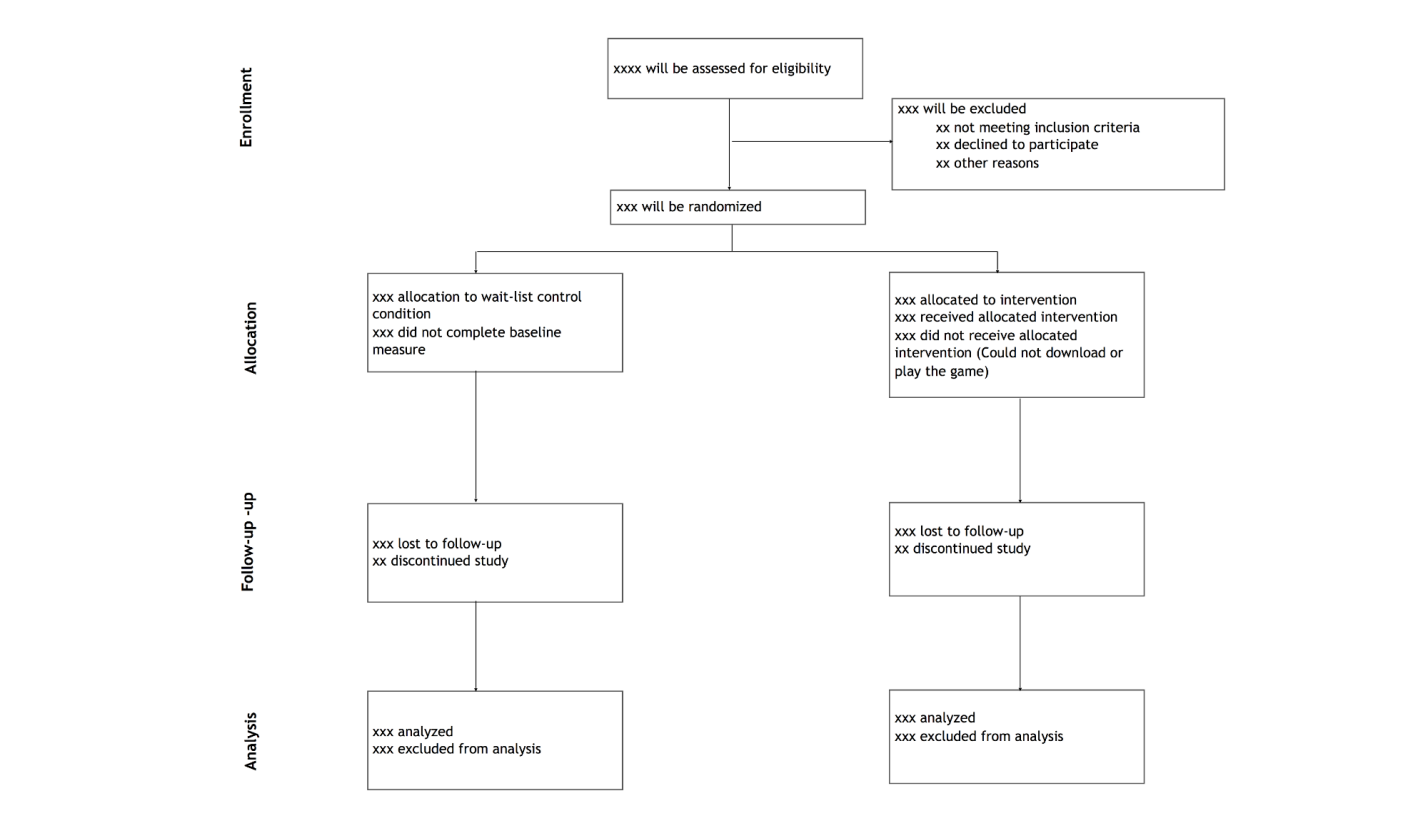
CONSORT diagram for Swaziland serious games-based trial.

The Swaziland Serious Games–Based HIV Prevention Trial (SGprev trial) will be a 4-week, two-arm randomized intervention trial. Participants will be randomized into 2 groups (the intervention group and a wait-list control group) in a 1:1 allocation ratio (Figure 1) [18]. The intervention will be downloadable from our website, on the Google Play store, and in popular cellular shops around Swaziland. These cellular shops already serve as distribution sites for other mobile software and apps, such as antiviruses, Opera mini, and WhatsApp, and are thus very popular in Swaziland. Additionally, the game will also be available from kiosks in all tertiary institutions in the country. Downloading the game will not be synonymous with enrolling in the trial. After downloading the game, potential participants will be redirected or prompted to visit our website where information about the trial and eligibility screening will be provided.

We plan to test whether a serious game intervention delivered on mobile phones to increase HIV risk perception, increases the intention to reduce multiple sexual partnerships, intention to know own HIV status, and intention to know all sexual partners’ HIV status will be more effective compared with current prevention efforts. Therefore, our hypotheses are that

1. The change in HIV risk perception between pre- and post-intervention assessment is greater in the intervention group compared with the change in the control group.

2. The change in HIV risk perception between pre- and post-intervention assessment will be greater among those reporting high HIV risk behavior in the intervention group compared with the control group.

3. The change in intention to have an HIV test between pre- and post-intervention assessment will be greater in the intervention group compared with the change in the control group.

4. The change in intention to reduce multiple concurrent partnerships between pre- and post-intervention assessment will be greater in the intervention group compared with the change in the control group.

5. The intervention group will report higher rate of condom use in the last sexual encounter compared with the control group.

### Participants, Setting, and Intervention

Our target population is Swazi males and females between 18- to 29-years old currently in Swaziland. Our intervention study targets people between 18- and 29-years old for the following reasons: (1) according to Bicego and colleagues [4], young people between this age group are most vulnerable to HIV because of their low HIV-testing behavior, (2) our primary study recruitment platform will be Facebook because the majority of mobile phone users in Swaziland also use Facebook, and (3) in our formative research (unpublished work, 2014) we found that this age group is likely to use smartphones and be literate on navigating the Internet compared with younger than 18-years-old or older than 29-years-old participants. Moreover, this age group is likely to find the SwaziYolo game entertaining.

### Inclusion Criteria

For this study, we will include males and females if they meet the following criteria: (1) are between 18 and 29 years of age, (2) own a smartphone running an Android-based operating system, (3) currently have the WhatsApp messaging app, (4) currently live in Swaziland, and (5) are able to adequately grant informed consent.

### Sampling Method

To recruit participants, we will post a targeted (based on our inclusion criteria), clickable banner advertisement on Facebook. After clicking on the advertisement, potential participants will be redirected to our website. Those who meet our eligibility criteria and have granted informed consent will be sent a unique trial verification code via text message and email. This unique trial verification code will be used to take our survey. Moreover, participants eligible for this trial will be entered into a lottery draw with a 1:100 chance of receiving US $20.

### Study Setting

The Kingdom of Swaziland, situated in Southern Africa, is a small land-locked country, the area of Swaziland is estimated to be 17,364 km^2^ with an estimated population of 1,146,050 (2006) [19]. According to Facebook there are currently approximately 160,000 people on Facebook, of those, 97,000 of them are man and women between the ages of 18- and 29-years old. Our primary recruitment site will be Facebook. Facebook is one of the most widely used social networking platforms in Swaziland and allows for targeted advertisements specifically to send people to our website. These two factors make Facebook an ideal platform to reach the Internet population in Swaziland. Participants do not need to be Facebook users to participate in the trial because our website can be assessed directly from the Internet. Participants will not be discouraged to share the study website link on other platforms, such as WhatsApp, Instagram, Email, and others.

### Description of the Intervention

SwaziYolo (a smartphone game) is an interactive, educational story game that puts the player in the role of a young adult looking for love in Mbabane (the capital city of Swaziland), making important choices about relationships and sexual health (see Multimedia Appendix 1 for an overview of the steps taken in developing the intervention). The intervention is guided by cognitive-based approaches such as the Theory of Planned Behavior to target intentions to engage in HIV protective behavior [20-22] and the Health Belief Model to target perceived susceptibility of acquiring HIV infection [20]. While capitalizing on elements of serious games such as immersion, role-playing, and a dynamic storyline, the game exists in two major parts: the first is set in an imaginary social network called SwaziYolo, which is meant to resemble a combination of existing tools like Facebook, OKCupid, and Whatsapp. Here, players register (registration and login screen), view pictures (potential love interests “Connections” screen) and profiles of potential love interests (profile mode screen), and have Web-based chats (chat mode screen) with various characters. The other half of the game takes place in various made-up locations around Mbabane, such as nightclubs and cafes (meet up at a club screen) where players regularly go on dates referred to in the game as “meet-ups.” In both parts of the game, players are regularly required to choose between several courses of action to progress a conversation or storyline with a friend or love interest (chat mode screen). The decisions they make will influence the opinions and behavior of other characters, as well as the player’s own health and safety. Eventually, feedback is given based on choices made (feedback from a medical doctor at a clinic screen). In the game, the various character dialogues and scenes, will address the issues identified in our formative research such as HIV risk perception, raising knowledge of their own HIV status as well as a sexual partner’s HIV status, reducing multiple concurrent sexual partnerships, and consistent condom use.

**Figure 2:**
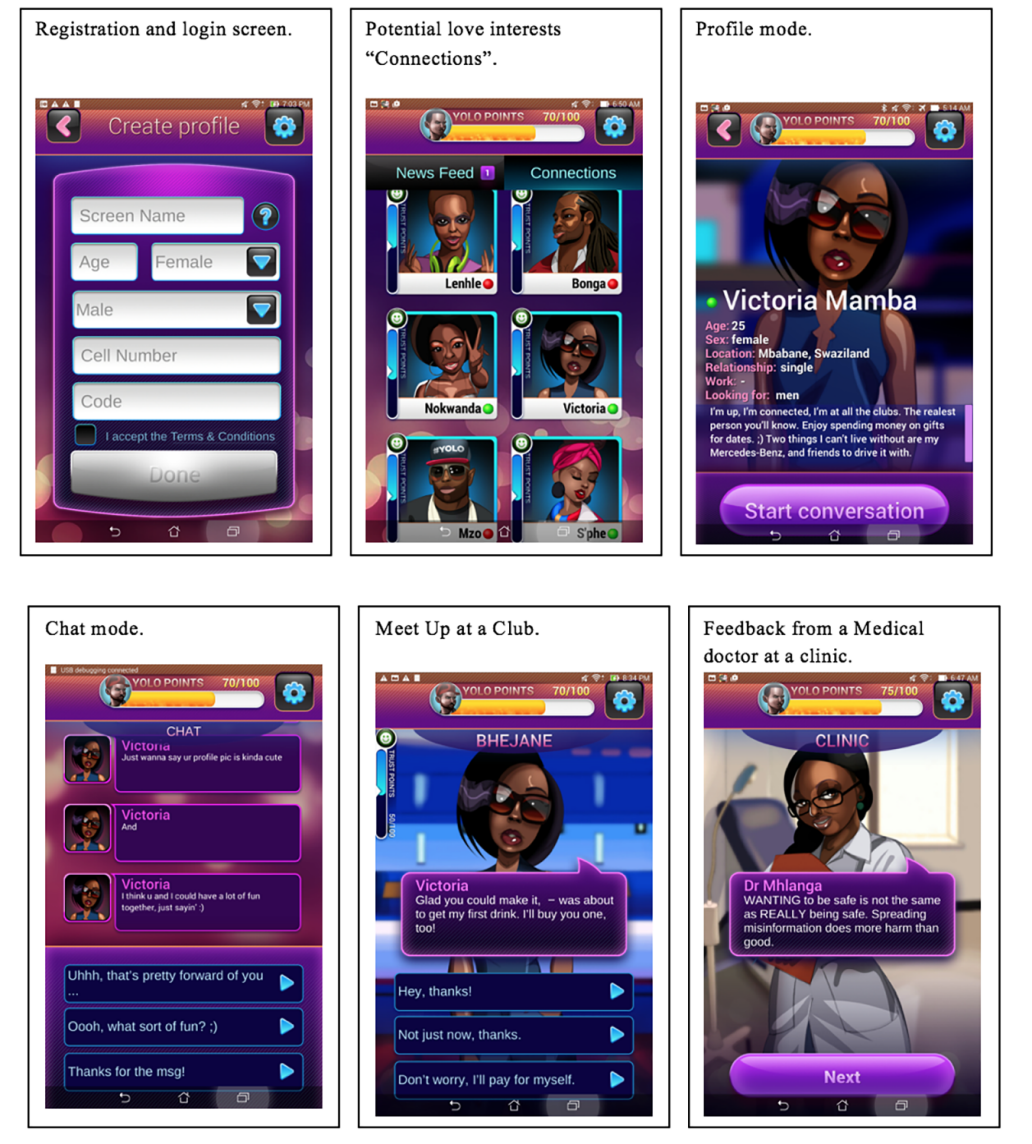
SwaziYolo screenshots.

The goal of the game is to maintain relationships with the characters, while staying healthy and happy. Once all the interactions with the characters have been completed, the game will give feedback on choices made and the risks those choices might carry. The game is expected to have an immense appeal to the youth, as an exciting new way to use their smartphones (see Figure 2 for the actual SwaziYolo screenshots). Participants in the wait-list control condition will complete the baseline and the immediate posttest measures as those in the SwaziYolo intervention condition, but will not play the game until the post-intervention assessment.

### Game Play

Player’s curiosity to “know what happens when they make a choice” is key to user engagement. The game’s narrative is primarily concerned with matters of sexual health, especially as it relates to HIV. Players will usually find themselves in situations where they have to make important decisions about their health, for example, resisting the pressure to have unprotected sex. The game keeps track of how well a player’s relationship is going with other characters using “TRUST” ratings (intimacy ratings), and a “YOLO” rating: how safe (safe relates to making choices that do not expose player to HIV risk) they have been during the course of the game. While players enjoy game play, they are exposed to valuable learning situations and are encouraged to care more about the various characters. Some will give good advice, while others will find themselves in difficult situations where they ask other players for help and guidance.

### Sample Size Calculation

The trial will be powered on the primary outcome measure and based on comparison of the change in HIV risk perception score from pre- to post-intervention assessment in the intervention group at 4 weeks. A study conducted in Uganda estimated mean HIV risk perception of M=3.27, SD *=* 1.03, therefore, we used this estimation as our baseline mean and SD to calculate our effect size [21]. Assuming a moderate effect size of 0.477 (identified by Chu et al [22]), alpha (two-tailed)=0.05, beta=0.20, with 1:1 allocation ratio between both groups, and a SD of the outcome in the population of 1.03, the total sample required to sufficiently power the study would be 146 (73 for each group). Moreover, assuming a 30% loss to follow-up rate [15], we then inflated the sample by 30% to yield a sample of 190. After that, we considered gender differences and once more inflated the sample by a factor of 2 to give a final sample size of 380. Finally, to ensure a balance between males and females in both groups we will recruit 190 females and 190 males. The sample size was calculated using Web-based sample size calculation software [23,24].

### Randomization

Upon confirmation of participants’ trial registration, participants will be assigned another unique code called “game unlock code,” which will be used for randomization using secure, remote, Web-based computer software within 24 hours of recruitment. As stated in the research design, participants will be randomized into 2 groups; the intervention group and the wait-list control group in a 1:1 ratio. The data analysis team will be blinded in this study, however, participants will not be blinded.

### Measurement Instrument

A self-administered structured Web-based questionnaire was created based on review of both Swazi and international literature. For example, questions relating to sociodemographic characteristics were adopted from the 2007 Swaziland Demographic Health Survey, and those related to risky sexual behavior and intention to change behavior were developed from our formative studies. Additionally, the Perceived Risk of HIV Infection Scale (PRHS; found to have excellent internal consistency Cronbach alpha=0.88) [11] will be used to assess the primary outcome of this study. Past research has used a variety of approaches to measure HIV risk perception including single likelihood assessments [21,25]. The 8-item PRHS scale incorporates items assessing cognitive assessments of risk (eg, chance of infection), as well as intuitive assessments (eg, feeling vulnerable, worry, gut feeling about likelihood), and salience of risk (eg, thought about risk, can picture it happening) to provide a more comprehensive measure of perceived risk of HIV infection, thus our choice to use this scale. The questionnaire will be converted into a Web-based format accessible via a link. Detailed variables assessed by the questionnaire are described in the section below and the questionnaire is presented in Multimedia Appendix 2. The trial tools were piloted among respondents known to the principal investigator, who will not be part of the main trial, in order to assess Web-based eligibility screening functionality; user verification; participant randomization functionality; questionnaire skip logic functionality; and the average length of time it takes to complete the questionnaire.

#### Primary Outcome Measure

Adding one or more comparison groups to a pre- and post-intervention assessment will result in a stronger intervention design than having a single intervention group to a pre- and post-intervention assessment [26]. Therefore, the primary intervention outcome will be the change in HIV risk perception score from pre- to post-intervention assessment in the intervention group compared with the change in the wait-list control group. High perception is considered to be the first stage toward behavioral change and has been associated with HIV protective HIV behavior [27,28]. HIV risk perception using the PRHS will be used to measure the primary outcome at baseline and at 4-weeks follow-up.

#### Secondary Outcomes Measure

The secondary outcomes for this intervention are self-reported intention to have an HIV test; intention to reduce multiple concurrent sexual partnerships; and an increase in reported condom use in the last sexual encounter (Table 1).

**Table 1:** Secondary outcome measures.

Measures		Baseline	Follow-up at 4 weeks
**Sexual reproductive history**			
	Condom use in the last sex	X^a^	X
	Number of sexual partners	X	X
**Intent to change behavior**			
	Intention to test for HIV and know partners’ HIV status	X	X
	Intention to reduce multiple concurrent partnerships	X	X
	Intention to use a condom	X	X
**Steady sexual partner’s history**			
	Has current sexual partner ever tested for HIV?	X	X
	Knowledge of current partner’s HIV status	X	X
**Demographics**			
	Age	X	
	Level of education	X	
	Employment status	X	
	Income level	X	
	Marital status	X	
	Ever tested for HIV	X	X
**Contact information**			
	Cellphone number and email	X	
**User experience**			
	Would you recommend the game to your friends?		X
	How did you hear about this game?		X
	Number of times player reached the end of the game		X
	Level of satisfaction about the game		X

^a^Timing of assessment.

### Data Collection Procedures

#### Baseline Data Collection

Participants will be recruited from Facebook users in Swaziland via a targeted Facebook advertisement. Potential participants will be directed to the study Web page where information about the intervention trial will be given and if willing, screening for eligibility will be done. After screening for eligibility, eligible individuals interested in participating in the trial will have an opportunity to ask detailed questions via free text message service offered by the WhatsApp app, Facebook messenger, or calling us. Sufficient time will be allowed for making an informed decision about participation in the study. Recruitment into this study will continue until our sample size is achieved.

After informed consent, a trial verification code will be sent to the participants via their mobile phones to prevent multiple identities in line with the CONSORT-EHEALTH guidelines 4a(ii) [29]. Upon confirmation of the unique verification code, participants will be enrolled in the trial and randomized into a control or intervention group. Subsequently, participants will be asked to answer the baseline Web-based questionnaire. In addition to study variables, contact information in the form of cellphone numbers will be collected at baseline to facilitate location of the research participants in the 1-month follow-up period [23,30]. The detailed questionnaire is outlined in Multimedia Appendix 2.

#### Four-Weeks Follow-Up Data Collection

Trial participants will be followed-up for 4 weeks, the game will collect log data and send this data to our servers when the participant goes on the Internet, this will allow us to assess the exposure to the intervention without over burdening our participants to manually send us their usage data. Data captured will be limited to login data. In addition to this, a Web-based questionnaire will be sent to participants at the end of the follow-up period. Participants who will miss their 4-week follow-up assessment will be actively traced though phone calls and text messages.

### Data Management and Statistical Analysis

#### Data Quality Assurance

First, Web-based questionnaires must be usable even for less experienced and knowledgeable Internet users [31], therefore we will exploit specific technical possibilities offered by open source Web-based questionnaires, such as visually highlighting buttons and predefined input fields. Additionally, we will use help features and input checks to assist participants when filling out the Web-based questionnaire. Beyond this, we will pilot test all filters and instructions given in the questionnaire. Second, to limit undesired multiple participation [32], either at baseline assessment or follow-up assessment, “sessions” will be used together with a verification code that participants will receive upon giving informed consent. Third, a specific problem that is faced by Web-based surveys is that respondents may “click through” the questionnaire, a phenomenon that becomes apparent when the interview is completed in less than the theoretical minimum time [31], therefore, the responses will be checked for plausibility and consistency and inconsistent records will be documented and censored from the final analysis.

#### Baseline Characteristics

Initially, descriptive statistics for the sample characteristics will be done for the intervention group and the wait-list control group to assess the distribution of important predictors of the outcome between both groups at baseline.

#### Primary Outcome Measure: HIV Risk Perception Score

First, we will use bivariate analysis to calculate the mean between baselines and follow-up. Next, to estimate the difference between the 2 groups, we will calculate the difference between the mean change of the intervention group and the mean change of the wait-list control group using two-sample paired *t* test. We will not perform interim analysis.

Secondly, in the case that, even after randomization, we observe some baseline differences, we will use multiple linear regression to adjust for those differences; where the outcome will be the follow-up score and the independent variables will be the intervention group, baseline scores, age, gender, marital status, level of education employment status, current monthly income, and number of times players played SwaziYolo. We will present our results in standardized regression coefficients for the intervention effect on the outcome variable as previously done for this type of hypothesis [25].

Although great effort will be put to minimize attrition, it is common for eHealth trials to typically have substantial attrition [26]. For this reason, our primary outcome analysis will prioritize analysis of the subjects who adhered to their group assignment and were sufficiently exposed to the intervention. Therefore, both pre-protocol analysis (as treated analysis) as well as intention to treat analysis will be done and both results will be reported. The approach of conducting both “intention to treat analysis” together with “as treated analysis” has been observed in literature for example, Weinstein and colleagues [33] followed this approach in their randomized trial comparing surgical versus nonoperative treatment for lumbar disk herniation.

#### Secondary Outcome Measures

Two-sample generalization McNemar’s test will be done to assess whether a significant change occurred between the pre- and the post-intervention assessments for dichotomous variables such as: intention to know self and partners HIV status, intention to reduce multiple sexual partners, and intention to use a condom the next time a participant has sexual intercourse. Each of these, outcomes will be assessed separately (individually). In order to judge the change, we will calculate the proportions of the dichotomous variable pre- and post-intervention in both groups. After that, we will obtain the pre- and post-intervention difference percentage at a *P* value within group and a *P* value in the intervention versus waitlist control group. This technique is documented by Katz [26]. Additionally, we will conduct a subgroup analysis of those with low-risk perception who report no condom use at last sexual encounter. This subgroup analysis will give us a more nuanced insight of the effect of the intervention to the most as risk subgroup in our study.

### Informed Consent

All participants will be required to give Web-based informed consent (Multimedia Appendix 3) before participation in the study. An online forum via Facebook and WhatsApp will be setup to allow participants to ask questions related to this research. They will be informed about the purpose of the study, its strict confidentiality, importance, and voluntary nature of their participation, their right to end the participation at any time without having to state a reason. Lastly, participants will be informed that the aggregated results (not individual case data) will be disseminated to improve the intervention package and general HIV prevention in Swaziland (see SGprev Trial information sheet in Multimedia Appendix 4).

### Protection of Personal Information

The following measures will be taken to protect participant’s personal information:

1. Permission will be sought from study participants to collect game usage data (login data) automatically.

2. All participants’ data will be stored under encrypted servers to protect participants’ information.

3. Participants’ cellphone numbers will be stored in a password protected file and will not be used for purposes other than those outlined in this protocol. After the trial, all cellphone numbers will be deleted.

### Expected Adverse Effects and Countermeasures

During or after the study, participants may develop psychological distress or embarrassment. All efforts to prevent this psychological distress or embarrassment will be put in place. If despite our efforts any psychological issue arises during the intervention and data collection, the research team will refer the participants’ to the nearest counselor (who is well vest on psychological issues) for appropriate psychological care and support via text messaging or calling. Participants will be encouraged to self-report any feelings of distress or discomfort to the research team using Web-based tools such as the WhatsApp app, Facebook private message, or via our contact details provided in the study information sheet including a toll-free number for HIV counseling.

### Data Storage

All data will be stored in the password-encrypted servers. Upon completion of the survey, all data will be exported to a password protected desktop computer at Kyoto University Department of Global Health and Socio-Epidemiology. Persons not part of the research team will not have any access to the collected data.

### Incentive

A lottery draw at baseline with a 1:100 chance of receiving US $20 will be given to all participants at the end of data collection as an incentive for their time in taking part in this trial. This amount was chosen carefully not to cause undue influence to the target population in that it is not excessive and is fair considering the country’s socioeconomic status.

## Results

### Current Status

The status of the study is in preinitiation stage. Results are expected in February 2017. We will present results as percentages, observed means with 95% confidence intervals, mean difference and 95% confidence intervals, standardized regression coefficients, and *P* values. All analysis will be performed using SPSS for Windows.

### Ethical Considerations

The study will be conducted according to the principles outlined in the Declaration of Helsinki International Guidelines for Ethical Review of Epidemiological Studies (CIOMS, 1991 Geneva). Furthermore, the research and development approval has been obtained from Kyoto University Graduate School and Faculty of Medicine Ethics Committee, Japan, and Swaziland’s Ministry of Health Ethics and Scientific committee. Caution will be taken to protect participant’s privacy during the data collection, data handling, and data reporting.

### Funding

Development of “SwaziYolo” serious game was funded by Hayao Nakayama Foundation for Science & Technology and Culture; Grant number H26-A2-41. The research implementation will be sponsored by the Department of Global Health and Socio-Epidemiology, Kyoto University, Japan.

## Discussion

### Overview

The risk of HIV infection is high among young people who practice risky sexual behavior, often they do not perceive their risk to be high, a phenomenon termed optimistic bias [11,27,28,34-36]. Some studies have reported that increased risk perception leads to subsequent increase in HIV protective behaviors, such as acceptance of HIV testing [37] or condom use [38]. The mechanism that increased perception leads to protective behavior is implicit in many behavioral theories as noted by Napper and colleagues [11]. Consequently, the joint United Nations Programme on HIV/AIDS (UNAIDS) guidance note on “social and behavior change programming” outlines risk perception as a thematic focus area for effective HIV prevention [39].

### Trial Implications

In line with the guidance form UNAIDS, this trial will provide a robust and rigorous evaluation of the efficacy of mobile serious games in increasing HIV risk perception in a resource limited setting such as Swaziland. Findings from this study will be made available to Swaziland authorities and stakeholders working to improve HIV prevention in Swaziland. We envision that the results of this study will be highly relevant to HIV prevention interventions in Swaziland and will inform future innovative strategies for HIV prevention. We are hopeful that our results will be generalizable to other settings in SSA. To our knowledge this is the first randomized control trial of a mobile serious games–based study to increase HIV protective behaviors in Swaziland and SSA; therefore, our findings will be a timely contribution to literature.

## Appendix

Multimedia Appendix 1 Intervention development.

Multimedia Appendix 2 Pre- and post-intervention questionnaire for the SGpriv trial.

Multimedia Appendix 3 Informed consent form.

Multimedia Appendix 4 Trial information sheet.

## Abbreviations

AIDS: acquired immune deficiency syndrome
eNSF: Extended National Multi-sectoral HIV/AIDS Framework for 2014–2018
HIV: human immunodeficiency virus
M: mean
PRHS: perceived risk of HIV infection scale
SSA: Sub-Saharan Africa
UNAIDS: United Nations Programme on HIV/AIDS
