# Supplementary material for: Efficacy of Mobile Serious Games in Increasing HIV Risk Perception in Swaziland: A Randomized Control Trial (SGprev Trial) Research Protocol
Source: JMIR Res Protoc. 2016 Nov 22;5(4):e224. doi: 10.2196/resprot.6543 (PMC5141336; doi:10.2196/resprot.6543)
Supplement: Multimedia Appendix 1 [file resprot_v5i4e224_app1.pdf]

Development of the intervention

Our intervention brought together perspectives from stakeholders, potential target audience and evidence from HIV related studies in Swaziland and Sub-Saharan Africa. In doing this, we followed an example from Brown and colleagues in their work on "Serious Games for Relationships and Sex Education: Application of an Intervention Mapping Approach to Development". Below we provide an overview of the steps and activities in the development of SwaziYolo intervention.

Step	Activities (2013 - 2016)
1. Formative assessment	• We established a working team made up of academic researchers in Kyoto University, Swaziland Ministry of Health Personnel, Swaziland based Non-Governmental Organization, Game Development Company in Cape Town, South Africa and a Game publisher in Japan.<br>• We specified overall goals of the intervention<br>• The Principal Investigator consulted with key informants from the internet service provider in Swaziland to assess the technological readiness of implementation of a mobile phone intervention<br>• Conducted literature search for current and past individual level HIV interventions and identified information needs<br>• Conducted literature search for current and past Serious Games<br>• Attended a conference on Serious Games to identify current trends in the field.<br>• Conduced face-to-face interviews with potential target audience
2. Set program objectives	• We stated performance objectives and program timelines<br>• Used quantitative and qualitative studies/interviews to segment our target population and gain insights into determinants of risky behaviors<br>• Identified important changeable determinants as perception of HIV risk and intention to conduct health behavior
3. Identified theory based methods and practical strategies	• We did not find one specific cognitive based theoretical framework that suited our changeable determinates therefore we used constructs from the Theory of Planned Behavior (Focusing on the "intent component") and the Health Believe model (Focusing on the perceived susceptibility).<br>• Identified intervention platform, on HTML based game or Mobile application and decision on mobile operating systems.<br>• A role-playing story based game was chosen as it provided the emersion that would allow our audience to learn through deciding the direction of the story and be exposed to consequences and feedback mechanisms embedded in the story direction.<br>• Point reward system was chosen to represent real life conflict between immediate gratification (loss in points) and the choice of the right action which delays gratification (gain in points).<br>• To ensure that the game addresses the change objectives, we linked key decision points in the game to our evaluation plan adapted from the Perceived Risk of HIV infection Scale.
4. Program plan	• Prepared documentation (game design concept note, and study protocol, terms and conditions of game use, promotion brief)<br>• Game characters were based on our target audience segmentation<br>• Storyline developed based on day-to-day life events in Swaziland.<br>• Language in the storyline developed to mirror target audience colloquial lingo.<br>• Music themes designed in line with scenes to draw on emotional engagement.<br>• Alpha and beta game testing and input made to address identified problems.
5. Program implementation	• Identified distribution network to promote adoption<br>• Finalized intervention outcomes<br>• Publishing game protocol to document trial plan and solicit early feedback<br>• Determined adoption and implementation strategies
6. Intervention evaluation	• Specified evaluation design, statistical plan and clear testable hypotheses

Arnab S, Dunwell I, Debattista K. Serious Games for Healthcare: Applications and Implications. Chapter 7 p135-166

Games Health J. Medical Information Science Reference; 2013;2(5):313–4. PMID: 26196931
