# Supplementary material for: Efficacy of Mobile Serious Games in Increasing HIV Risk Perception in Swaziland: A Randomized Control Trial (SGprev Trial) Research Protocol
Source: JMIR Res Protoc. 2016 Nov 22;5(4):e224. doi: 10.2196/resprot.6543 (PMC5141336; doi:10.2196/resprot.6543)
Supplement: Multimedia Appendix 2 [file resprot_v5i4e224_app2.pdf]

Appendix I Questionnaire
Research Title:
Effectiveness of serious games in increasing HIV risk perception in Swaziland: A Randomized Control Trial (SGprev Trial)

Pre and post intervention questionnaire for the SGpriv Trial		
**#**	**QUESTION**	**RESPONSE**
**Socio Demographic Information**		
	What is your mobile phone number (participants will be asked this question during registration into the trial)	________________________
Q1	How old are you?	☐
Q2	What is your marital status?	☐<br>1. Single (Never married and not living with a partner)<br>2. Married :<br>3. Living with a partner<br>4. Separated (currently not living together but not divorced<br>5. Divorced<br>6. Widowed
Q3	What is your current highest level of education?	1. None<br>2. Primary level<br>3. Secondary level<br>4. High School level<br>5. Tertiary level ☐
Q4	What is your current employment status?	1. Employed<br>2. Not employed<br>3. Student<br>4. Self employed ☐
Q5	What is your (personal) current total monthly income from all sources?<br><br>If no income please select ⓪	1. Less than E249<br>2. More than E249 but less than E1749<br>3. More than E1749 but less than E3000<br>4. More than 3000 ☐
Q6	Have you ever had an HIV test	1. Yes<br>2. No ☐

Sexual reproductive history<br><br>The following section relates to questions about sexual activity in order to gain a better understanding of some reproductive health issues. Once more we would like to assure you of the confidentially and anonymous nature of this survey.		
Q7	Did you use a condom in your last sexual activity?	1. Yes①<br>2. No ☐

`

Q8	When was the last time you had sex without a condom	1. Within the past 30 days<br>2. 2-6 months ago<br>3. 7-12 months ago<br>4. More than 1 year ago
Q9	How many sexual partners did you have in the past 30 days?	

Thoughts about likelihood of getting HIV

This section relates to what you think are your changes of getting HIV infection (PRHS)

Q10	What is your gut feeling about how likely you are to get infected with HIV?	1. Extremely unlikely<br>2. Very unlikely<br>3. Somewhat likely<br>4. Very likely<br>5. Extremely likely
Q11	I worry about getting infected with HIV	1. None of the time<br>2. Rarely<br>3. Some of the time<br>4. A moderate amount of time<br>5. A lot of the time<br>6. All of the time
Q12	Picturing myself getting HIV is something I find	1. Very hard to do<br>2. Hard to do<br>3. Easy to do<br>4. Very easy to do
Q13	Getting HIV is something I am…	1. Not concerned about<br>2. A little concerned about<br>3. Moderately concerned about<br>4. Concerned about a lot<br>5. Extremely concerned about
Q14	I am sure I will NOT get HIV infected	1. Stonly disagree<br>2. Disagree<br>3. Somewhat agree<br>4. Somewhat disagree<br>5. Agree<br>6. Strongly agree
Q15	I feel I am unlikely to get infected with HIV	1. Stonly disagree<br>2. Disagree<br>3. Somewhat disagree<br>4. Somewhat agree<br>5. Agree<br>6. Strongly agree
Q16	I feel vulnerable to HIV infection	1. Stonly disagree<br>2. Disagree<br>3. Somewhat disagree<br>4. Somewhat agree<br>5. Agree<br>6. Strongly agree
Q17	There is a chance, no matter how small, I could get HIV	1. Stonly disagree<br>2. Disagree<br>3. Somewhat disagree<br>4. Somewhat agree<br>5. Agree<br>6. Strongly agree

`

Q18	I think my chances of getting infected with HIV are	1. Zero<br>2. Almost zero<br>3. Small<br>4. Moderate<br>5. Large<br>6. Very Large
Q19	Getting HIV is something I have	1. Never thought about<br>2. Rarely thought about<br>3. Thought about some of the time<br>4. Thought about often
**Intention to change your sexual behabour**<br>This section asks about your intention to change your sexual behabour in the coming months		
Q20	Have you ever been tested for HIV?	1. Yes<br>2. No
Q21	When were you last tested for HIV?	1. Within the past 30 days<br>2. 2-6 months ago<br>3. 7-12 months ago<br>4. More than 1 year ago
Q22	Do you intend to test for HIV in the coming months?	1. Yes<br>2. No<br>3. No I am alredy HIV Positive
Q23	Do you intend to know ALL your sexual partners' HIV statuses?	1. Yes<br>2. No
Q24	Do you intend to reduce your number of sexual partners in the next months?	1. Yes<br>2. No
Q25	Do you Intend to use a condom in the next time you have sexual intercourse	1. Yes<br>2. No<br>3. I don't know
This section asks about your sexual partner's history (current partner/s) if more than one partners then the last partner you had sexual intercourse with		
Q26	Has your stready partner ever tested for HIV	1. Yes<br>2. No
Q27	Do you know your steady partner's HIV status	1. Yes<br>2. No
SwaziYolo game (this section will be in pre and post survey)		

`

Q28	How did you hear about this game	1. Facebook<br>2. Times of Swaziland<br>3. Swazi Observer<br>4. Friend<br>5. Other (Spedicy)	☐

Experience with the SwaziYolo game

This next section asks about your experience with the SwaziYolo game (this questions will only be asked at post intervetion only to those who played SwaziYolo)

Q29	How many times did you play SwaziYolo from start to end ?	1. Once but did not get to the end<br>2. Once and I got to the end<br>3. Two times and I got to the end in both times<br>4. More than two times	☐
Q30	I would recommend the SwaziYolo game to my friends	5. Strongly agree<br>6. Agree<br>7. Disagree<br>Strongly disagre	☐
Q31	On a scale 1 – 5 (one being the list and 5 being the most) how satisfied are you with this game		☐
Q32	How did you hear about this game	1. Facebook<br>2. Times of Swaziland<br>3. Swazi Observer<br>4. Friend<br>5. Other (Spedicy)	☐

Pre-Intervetion closing message
Thank you for taking part in this survey, you may now continue to play the SwaziYolo game. Please click here to download the game and you will use this code ***** to play the game. (will be seen only by those randomised to the intervention group)

Pre-Intervetion closing message
Thank you for taking part in this survey, you have been sellected to be in the waiting list. You will be contacted after fourweeks to play the SwaziYolo game. (will be seen only by those randomised to the wait-list control group)

Post intervention closing message
Thank you for taking part in this survey you will be informed when the latest update of the SwaziYolo game in available. (will be seen only by those randomised to the intervention group)

Post intervention closing message
Thank you for taking part in this survey you may now continue to play the SwaziYolo game. Please click here (will be seen only by those randomised to the wait-list control group)
