# Supplementary material for: Efficacy of Mobile Serious Games in Increasing HIV Risk Perception in Swaziland: A Randomized Control Trial (SGprev Trial) Research Protocol
Source: JMIR Res Protoc. 2016 Nov 22;5(4):e224. doi: 10.2196/resprot.6543 (PMC5141336; doi:10.2196/resprot.6543)
Supplement: Multimedia Appendix 3 [file resprot_v5i4e224_app3.pdf]

Participant consent form

Survey:

Efficacy of serious games in increasing HIV risk perception in Swaziland: A randomized intervention trial (SGprev Trial)

Please read the following:

I confirm I have read and understood the information sheet for the above study and have had the opportunity to ask questions online.

I understand that my participation is voluntary and that I am free to withdraw at any time without penalty.

I freely agree to participate in this study.

Clicking the “Yes” below is a confirmation of the above and your explicit informed concern.

Yes

No
