# Supplementary material for: Efficacy of Mobile Serious Games in Increasing HIV Risk Perception in Swaziland: A Randomized Control Trial (SGprev Trial) Research Protocol
Source: JMIR Res Protoc. 2016 Nov 22;5(4):e224. doi: 10.2196/resprot.6543 (PMC5141336; doi:10.2196/resprot.6543)
Supplement: Multimedia Appendix 4 [file resprot_v5i4e224_app4.pdf]

Appendix III

STUDY INFORMATION SHEET

Study

Efficacy of mobile serious games in increasing HIV risk perception in Swaziland: A randomized intervention trial (SGprev Trial)

About the study and it's Purpose

Good morning/afternoon. My name is Bhekumusa Lukhele. I am a student at Kyoto University. We are conducting a survey to explore whether a serious game intervention delivered on mobile phones to increase HIV risk perception among mobile phone users in Swaziland is effective. We will have two groups for which membership is chosen at random by a computer system; Group one will take the survey then play a mobile phone game called SwaziYolo, and after 4 weeks they will be asked to retake the same survey. Group two will also take the survey twice but will wait until everyone in group one has finished playing the game then they will be given access to the game after taking the second survey. The purpose of this survey is to compare HIV risk perceptions between people who will play SwaziYolo game and those who will be waiting to play the game.

(See figure below for simplified representation)

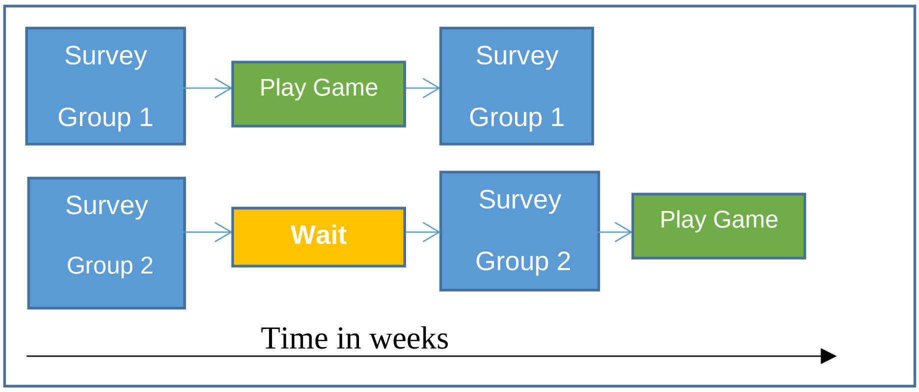

Risks

There are no direct health risks in participating in this intervention; however you might lose your airtime as you take this survey or download the game. Additionally the game will send usage data automatically for example: time of login, duration of play but no personal data will be automatically collected. The game is 49MB and available free of cost to the study participants. If there are words you do not understand please feel free to contact us at the details bellow for clarifications.

Benefits

You may benefit in learning about HIV prevention. Additionally you will have a 1 in 100 chance of winning US $20 (Emalangeni 200) gift voucher. This means that for every 100 people who will take our survey only 1 person will get (Emalangeni 200) gift voucher

Confidentiality

The information gathered in the survey will be kept strictly confidential; after the study is complete we will report aggregated data for example: 20 people out of 100 were male and not your individual information. Identifying information such as your mobile phone number will be collected however it will be delinked from your name or surname. We will use this contact information to contact you if a need arises for example if you won the rotary draw or we think you may have forgotten to take the survey at four weeks. After completion of the study we will delete all contact information.

Compensation

There will be no compensation for participating in this study except for the rotary draw where you will stand a 1 in 100 chance of winning a US $20 (Emalangeni 200) gift voucher. This means that for every 100 people who will take our survey only 1 person will get (Emalangeni 200) gift voucher. Therefore not everyone will win. The rotary draw winner will be selected by chance.

Voluntary Participation

You are free to either participate or not participate in this study. However, your participation is valuable as your views will contribute to the development of strategies to improve HIV prevention in Swaziland. You will find information about how to download the game in our website. The survey may take 10 to 15 minutes to complete and the playing the game may take 10 to 20 minutes per day. You are free to decline to answer any particular question or withdraw from the study at any time without penalty.

Who to contact if a need arise: for example if you are experiencing some discomfort due to taking this study or you would like to received more information.

Toll free number for counselling:
977

For questions related to the research
Facebook/swaziyolo
WhatsApp number:
Tel: +268 76239141

For Ministry of Health
Babazile Shongwe
Ministry of Health
Swaziland Ethics Committee secretariat
PO Box 5
Tel: 2404 7345

Bhekumusa Lukhele
Principal Investigator
Department of Global Health and Socio-epidemiology
Kyoto University School of Public Health
Tel: +81 75-753-4350
